# Supplementary material for: From Bird Viremia to Bird Surveillance: Identifiability in a Multiscale Vector-Borne Model of Usutu Virus Infection
Source: bioRxiv. 2025 Nov 17:2025.11.17.688793. Preprint. [Version 1] doi: 10.1101/2025.11.17.688793 (PMC12667886; doi:10.1101/2025.11.17.688793)
Supplement: Supplement 1 [file NIHPP2025.11.17.688793v1-supplement-1.pdf]

bioRxiv preprint doi: https://doi.org/10.1101/2025.11.17.688793; this version posted November 17, 2025. The copyright holder for this preprint (which was not certified by peer review) is the author/funder, who has granted bioRxiv a license to display the preprint in perpetuity. It is made available under aCC-BY 4.0 International license.

Supporting information

S1. Numerical Scheme In this section, we introduce a finite difference method for solving the time-since-infection model Eqs. (2.3)- (2.4). We begin by constructing the numerical mesh by discretizing the domain $D = \{(t, \tau) : 0 \leq t \leq M_t, 0 \leq \tau \leq M_\tau\}$, where τ is the infection age, t is epidemic time, M_t is the maximum time, and M_τ is the maximum infection age. We discretize the spans of infection age and epidemic time into subintervals such that $\Delta\tau = \kappa\Delta t$. Thus, the points in the epidemic time and infection age directions are given by $t_n = n\Delta t$ and $\tau_k = k\Delta\tau$, respectively, with $M_t = N\Delta t$, $M_\tau = K\Delta\tau$, and N and K the total number of subintervals. Let $i^n_{h_k}$ be the approximation of i_h at the point (t_n, τ_k). We denote the approximate solutions of the state variables at time t_n as S^n_v, I^n_v, S^n_h, and R^n_h. We first discretize the equation for the susceptible mosquitoes, S_v, by replacing the time derivative with a backward difference, and obtain:

$$\frac{S^{n+1}_v - S^n_v}{\Delta t} = \mu_v - S^{n+1}_v \sum_{k=1}^{K} \beta_v(\tau_k) i^{n+1}_{h_k} \Delta t - \mu_v S^{n+1}_v,$$

Next, we linearize the nonlinear term by evaluating i_h at epidemic time t_n instead of t_{n+1}, then solve for S^{n+1}_v to obtain:

$$S^{n+1}_v = \frac{S^n_v + \mu_v \Delta t}{1 + \Delta t \sum_{k=1}^{K} \beta_v(\tau_k) i^n_{h_k} \Delta t + \mu_v \Delta t}. \tag{4.1}$$

Similarly, we discretize the equations for the infected mosquitoes, I_v, and susceptible birds, S_h, by evaluating them at t_{n+1} and applying a backward difference. This process yields the following equations:

$$I^{n+1}_v = \frac{I^n_v + \Delta t S^{n+1}_v \sum_{k=1}^{K} \beta_v(\tau_k) i^n_{h_k} \Delta t}{1 + \mu_v \Delta t}. \tag{4.2}$$

$$S^{n+1}_h = \frac{S^n_h}{1 + \beta_h I^{n+1}_v \Delta t}. \tag{4.3}$$

Finally, we discretize the PDE, evaluating it at t_{n+1} and τ_k. We replace the derivative in τ with a forward difference and the derivative in t with a backward difference. This results in the

bioRxiv preprint doi: https://doi.org/10.1101/2025.11.17.688793; this version posted November 17, 2025. The copyright holder for this preprint (which was not certified by peer review) is the author/funder, who has granted bioRxiv a license to display the preprint in perpetuity. It is made available under aCC-BY 4.0 International license.

following difference equation:

$$\frac{i_{h_k}^{n+1} - i_{h_k}^{n}}{\Delta t} + \kappa \frac{i_{h_{k+1}}^{n+1} - i_{h_k}^{n+1}}{\Delta \tau} = -\gamma_h i_{h_{k+1}}^{n+1}.$$

Since $\dfrac{\kappa}{\Delta \tau} = \Delta t$,

$$\frac{i_{h_{k+1}}^{n+1} - i_{h_k}^{n}}{\Delta t} = -\gamma_h i_{h_k}^{n+1}.$$

To transform this method into an implicit scheme, we replace the term $i_{h_k}^{n+1}$ with $i_{h_{k+1}}^{n+1}$, and solve for $i_{h_{k+1}}^{n+1}$ and obtain:

$$i_{h_{k+1}}^{n+1} = \frac{i_{h_k}^{n}}{1 + \gamma_h \Delta t}. \tag{4.4}$$

Considering the boundary condition at $t = t_{n+1}$, we have,

$$\kappa i_{h_0}^{n+1} = \beta_h S_h^{n+1} I_v^{n+1}. \tag{4.5}$$

Given that the total bird population $S_h(t) + I_h(t) + R_h(t) = 1$ remains constant:

$$R_h^{n+1} = 1 - (S_h^{n+1} + I_h^{n+1}), \tag{4.6}$$

where $I_h^{n+1} = \sum_{k=1}^{K} i_{h_k}^{n+1} \Delta t$. Therefore, the finite difference problem, derived from Eqs. (4.1)-(4.6) becomes:

$$= \begin{cases} S_v^{n+1} = \dfrac{S_v^n + \mu_v \Delta t}{1 + \Delta t \sum_{k=1}^{K} \beta_v(\tau_k) i_{h_k}^n \Delta t + \mu_v \Delta t}, & n = 0, ..., N-1, \\ I_v^{n+1} = \dfrac{I_v^n + \Delta t S_v^{n+1} \sum_{k=1}^{K} \beta_v(\tau_k) i_{h_k}^n \Delta t}{1 + \mu_v \Delta t}, & n = 0, ..., N-1, \\ S_h^{n+1} = \dfrac{S_h^n}{1 + \beta_h I_v^{n+1} \Delta t}, & n = 0, ..., N-1, \\ i_{h_{k+1}}^{n+1} = \dfrac{i_{h_k}^n}{1 + \gamma_h \Delta t}, & n = 0, ..., N-1, k = 0, ..., K-1 \\ i_{h_0}^{n+1} = \dfrac{\beta_h}{\kappa} S_h^{n+1} I_v^{n+1}, & n = 0, ..., N-1, \\ R_h^{n+1} = 1 - (S_h^{n+1} + I_h^{n+1}), & n = 0, ..., N-1. \end{cases} \tag{4.7}$$

bioRxiv preprint doi: https://doi.org/10.1101/2025.11.17.688793; this version posted November 17, 2025. The copyright holder for this preprint (which was not certified by peer review) is the author/funder, who has granted bioRxiv a license to display the preprint in perpetuity. It is made available under aCC-BY 4.0 International license.

S2. Monte Carlo simulation results Practical identifiability results are presented below. 575

Parameter	β	k	δ	π	c
ARE					
$\sigma = 1\%$	0.1	0	0	0	0
ARE					
$\sigma = 5\%$	1.3	0.2	0.4	1.3	0.8
ARE					
$\sigma = 10\%$	3.5	0.4	1.7	3.3	1.7
ARE					
$\sigma = 20\%$	34.6	6.5	11.1	25.9	24.9

Table 10. MCS results for the within-host model: Monte Carlo simulation results for virtual datasets generated at each data point in Table 3. The AREs give the average relative estimation errors for each parameter of the within-host model (Eq. (2.1)) at noise level σ.

Parameter	a	h
$\sigma = 1\%$		
ARE	10	1.6
$\sigma = 5\%$		
ARE	54.9	8.2
$\sigma = 10\%$		
ARE	119.4	17.7
$\sigma = 20\%$		
ARE	197.1	35.2

Table 11. MCS results for the per-bite percent mosquito infection model: Monte Carlo simulation for virtual datasets generated at each data point in Table 4. The AREs give the average relative estimation errors for each parameter of the probability of infection function Eq. (2.2) at noise level σ.

bioRxiv preprint doi: https://doi.org/10.1101/2025.11.17.688793; this version posted November 17, 2025. The copyright holder for this preprint (which was not certified by peer review) is the author/funder, who has granted bioRxiv a license to display the preprint in perpetuity. It is made available under aCC-BY 4.0 International license.

Parameter	β_h	c_v	γ_h	μ_v
ARE				
$\sigma = 1\%$	2.8	4.1	16.8	1.2
ARE				
$\sigma = 5\%$	16.9	31	75.7	6.3
ARE				
$\sigma = 10\%$	30.4	65.1	136.5	10.7
ARE				
$\sigma = 20\%$	51.1	99.1	166.6	18.5

Table 12. MCS results for the between-host model: Monte Carlo simulation results for virtual datasets generated at each data point in Table 5. The AREs give the average relative estimation errors for each parameter of the between-host model given in Eqs. (2.3)-(2.4) at noise level σ.

Parameter	β_h	c_v	γ_h	μ_v	β	k	δ	π	c
ARE									
$\sigma = 1\%$	0.4	0	16.8	0.6	0.9	2.3	3.9	13.5	4.2
ARE									
$\sigma = 5\%$	1.5	0	47.8	2.4	3.2	0.6	1.4	6	2
ARE									
$\sigma = 10\%$	4.4	0	84.2	6.3	7.5	0.7	1.7	9.9	3.6
ARE									
$\sigma = 20\%$	12.6	0	146.3	14.1	17.7	2.7	4	19	7.1

Table 13. MCS results for the multiscale model: Monte Carlo simulation results for virtual datasets generated at each data point in Tables 3 and 5. The AREs give the average relative estimation errors for each parameter of the multiscale model given in Eqs. (2.1)-(2.4) at noise level σ.
